# Lightweight rice leaf spot segmentation model based on improved DeepLabv3+

**DOI:** 10.3389/fpls.2025.1635302

**Published:** 2025-08-22

**Authors:** Jianian Li, Long Gao, Xiaocheng Wang, Jiaoli Fang, Zeyang Su, Yuecong Li, Shaomin Chen

**Affiliations:** ^1^ Faculty of Modern Agricultural Engineering, Kunming University of Science and Technology, Kunming, China; ^2^ Faculty of Information Engineering and Automation, Kunming University of Science and Technology, Kunming, China

**Keywords:** rice leaf diseases 1, segmentation 2, DeepLabV3 + 3, light-weight model 4, feature fusion 5

## Abstract

**Introduction:**

Rice is an important food crop but is susceptible to diseases. However, currently available spot segmentation models have high computational overhead and are difficult to deploy in field environments.

**Methods:**

To address these limitations, a lightweight rice leaf spot segmentation model (MV3L-MSDE-PGFF-CA-DeepLabv3+, MMPC-DeepLabv3+) was developed for three common rice leaf diseases: rice blast, brown spot and bacterial leaf blight. First, the lightweight feature extraction network MobileNetV3_Large (MV3L) was adopted as the backbone of the model. Second, based on Haar wavelet downsampling, a multi-scale detail enhancement (MSDE) module was proposed to improve decision-making ability of the model in transitional regions such as spot gaps, and to improve the sticking and blurring problems at the boundary of spot segmentation. Meanwhile, the PagFm-Ghostconv Feature Fusion (PGFF) module was proposed to significantly reduce the computational overhead of the model. Furthermore, coordinate attention (CA) mechanism was incorporated before the PGFF module to improve robustness of the model in complex environments. A hybrid loss function integrating Focal Loss and Dice Loss was ultimately proposed to mitigate class imbalance between disease and background pixels in rice disease imagery.

**Results:**

Validated on rice disease images captured under natural illumination conditions, the MMCP-DeepLabv3+ model achieved a mean intersection over union (MIoU) of 81.23% and mean pixel accuracy (MPA) of 89.79%, with floating-point operations (Flops) and the number of model parameters (Params) reduced to 9.695 G and 3.556 M, respectively. Compared to the baseline DeepLabv3+, this represents a 1.89% improvement in MIoU, a 0.83% increase in MPA, alongside 93.1% and 91.6% reductions in Flops and Params.

**Discussion:**

The MMPC-DeepLabv3+ model demonstrated superior performance over DeepLabv3+, U-Net, PSPNet, HRNetV2, and SegFormer, achieving an optimal balance between recognition accuracy and computational efficiency, which establishes a novel paradigm for rice lesion segmentation in precision agriculture.

## 1 Introduction

As the global leader in rice cultivation and consumption, China maintains an annual production area of approximately 30 million hectares with consistent yield outputs exceeding 200 million metric tons (Chen et al., 2025). Phytopathological threats during vegetative growth stages, however, persistently compromise both yield potentials and grain quality indices. Historically, disease identification was primarily reliant on empirical farmer observations (Sethy et al., 2020), a methodology constrained by diagnostic subjectivity, operational inefficiency, and limited classification accuracy. With the rapid development of computer technology, traditional agriculture is gradually transforming to modern agriculture, and computer vision technology can more accurately and efficiently identify and localize diseases on rice leaves (Tian et al., 2020).

Current crop disease lesion segmentation based on computer vision techniques is primarily divided into two research directions: traditional image processing algorithms and deep learning approaches. However, the former methodology is constrained by its reliance on manually predefined feature extractors, predefined features may become invalid when confronted with phenotypic variations in disease manifestations, leading to significant performance degradation of systems. More critically, these algorithms exclusively operate on low-level visual features, which exhibit limited discriminative capacity in scenarios involving complex field backgrounds or low lesion-background chromatic contrast.

In contrast to traditional image processing algorithms, Convolutional Neural Network (CNN)-based segmentation models are capable of autonomously extracting hierarchical features from raw image data, thereby enabling end-to-end disease lesion segmentation. Notably, with increasing network depth, the extracted features progressively transition from low-level visual cues to semantic-rich high-level representations. This hierarchical feature learning mechanism not only improves the semantic understanding of pathological patterns in the model but also enhances robustness to complex agricultural field environments. Therefore, CNN-based approaches have emerged as the dominant methodology for crop disease lesion detection in recent literature.

To address the critical need for improving detection accuracy and operational efficiency in agricultural disease management, CNN-based crop lesion segmentation models have been widely researched and refined by global researchers. Ren et al. (2020) extracted features of tomato leaf diseases using VGG16, utilized skip connections in the decoder to fuse multiple features and restore image details, and finally upsampled to the input size through transposed convolution to achieve the segmentation of tomato disease spots and background. The mean intersection over union (MIoU) and mean pixel accuracy (MPA) reached 75.36% and 94.66% respectively. Li et al. (2023) proposed the MA-Unet model based on the Unet network for segmenting cucumber disease spots and healthy leaves. By incorporating hybrid dilated convolution blocks into the encoder of the Unet network to enlarge the receptive field, and embedding the Convolutional Block Attention Module (CBAM) in the decoder to reduce interference from complex backgrounds, precise segmentation of disease spots and healthy leaves was achieved. The MIoU index reached 84.97%. Wang et al. (2023) addressed the challenges of small lesion areas and blurred boundaries in pear tree leaf diseases by integrating a multi-feature extraction module and dynamic sparse attention mechanism into the Unet model. This integration further improved the ability of the model to capture global features, ultimately achieving accurate segmentation among disease spots, healthy leaves, and background regions. The MIoU reached 86.15%. Wang et al. (2021) designed a cascaded DUNet model combining DeepLabv3+ and Unet to address the poor segmentation of cucumber disease spots under complex backgrounds. By calculating the proportion of diseased areas to healthy leaves, the model achieved the determination of cucumber disease severity. Convolutional neural networks can automatically learn complex feature representations from large-scale data, reducing reliance on specialized domain knowledge while demonstrating better adaptability and robustness under changing environmental conditions.

Recently, Fu et al. (2024) enhanced the segmentation accuracy of pear leaf diseases using an improved DeepLabv3+ architecture integrated with MobileNetV2 and SE attention, demonstrating the feasibility of lightweight and attention-enhanced networks in agricultural pathology applications. Pal et al. (2025) proposed a MAML-based DeepLabv3 framework integrating multi-scale spatial attention, enabling both lesion segmentation and disease severity assessment with minimal computational overhead, further underscoring the relevance of attention mechanisms in agricultural diagnostics. In addition, Mo et al. (2022)demonstrated that combining coordinate attention with CBAM within a DeepLabv3+ framework significantly enhanced lesion boundary detection while retaining model efficiency. These studies collectively affirm the value of lightweight architectures and attention-based designs, which align with the goals of our work.

While CNN-based crop disease lesion segmentation has become the current mainstream approach and achieved substantial progress, there remain the following challenges: (1) Most existing studies mainly focus on distinguishing lesion areas from healthy leaves or backgrounds, failing to precisely differentiate different disease types. (2) Existing lesion segmentation models typically feature complex architectures with large parameter sizes and high computational costs. While these models perform excellently on high-performance computing devices, they show poor performance when deployed in real agricultural environments with limited computational resources. Therefore, there is an urgent need to develop a more targeted rice lesion segmentation model to address these issues. To tackle these challenges, this study focuses on three common rice diseases during growth: bacterial leaf blight, rice blast, and brown spot:

First, four mainstream crop disease segmentation models were trained and tested to obtain loss changes and performance metrics on the training set, confirming the selection of DeepLabv3+ as the baseline network for rice leaf lesion segmentation in this study. Next, to address the high computational cost of the DeepLabv3+ model, the Xception network used for feature extraction in the encoder was replaced with an MobileNetV3_Large (MV3L) network incorporating dilated convolutions. Secondly, a PagFm-Ghostconv Feature Fusion (PGFF) module and multi-scale detail enhancement (MSDE) module were proposed and introduced, enabling the model to maintain feature extraction capabilities while significantly reducing the number of model parameters (Params) and floating-point operations (Flops) of the model. Thirdly, the coordinate attention (CA) mechanism was introduced into the decoder to improve the robustness of the model in segmenting complex scenarios. Fourthly, to address the imbalance in the proportion of different parts in rice disease images, a hybrid loss function combining Focal Loss and Dice Loss was proposed.

## 2 Materials and methods

### 2.1 Dataset construction

The rice disease dataset used in this study was compiled from two primary sources. The first source consisted of 400 rice leaf images with natural complex backgrounds, including 100 samples for each category (healthy leaves, brown spot, rice blast, and bacterial blight), which were captured using a Huawei Mate50 smartphone at a rice cultivation base in Kunming, Yunnan Province. The second source comprised publicly available rice disease datasets obtained from Kaggle, containing 350 additional images per class. These included both complex-background and simple-background scenarios, specifically: brown spot (282 complex, 68 simple), rice blast (150 complex, 200 simple), bacterial blight (144 complex, 206 simple), and healthy leaves (185 complex, 165 simple).

To ensure dataset reliability, a two-stage quality validation process was applied. First, all images and their disease annotations were reviewed and confirmed by experienced agronomy experts to ensure biological accuracy. Second, all images were manually screened to eliminate those severely blurred or distorted to the extent that disease features were unrecognizable to the human eye.

In total, the original dataset contained 1,800 images evenly distributed across the four disease types and healthy leaves (450 per class). The dataset was split into a training set and a validation set at a 9:1 ratio. To improve the robustness and generalization ability of the model, we employed an online data augmentation strategy exclusively during the training stage. A flexible augmentation pipeline was constructed, in which each operation—including geometric transformations (e.g., rotation, flipping, scaling), color adjustments (brightness, hue, saturation), and noise injection (Gaussian and salt-and-pepper noise)—was applied with a specified probability during each training iteration. This approach dynamically diversifies the input data, reduces the risk of overfitting, and simulates diverse field conditions such as lighting changes, leaf deformation, and partial occlusion. Notably, this strategy has been validated in similar plant disease segmentation tasks. For example, Jia et al. (2024) applied an almost identical dynamic augmentation method in their DFMA-based segmentation framework and demonstrated significant improvements in performance and generalization under real-world agricultural environments.

**Figure 1:**
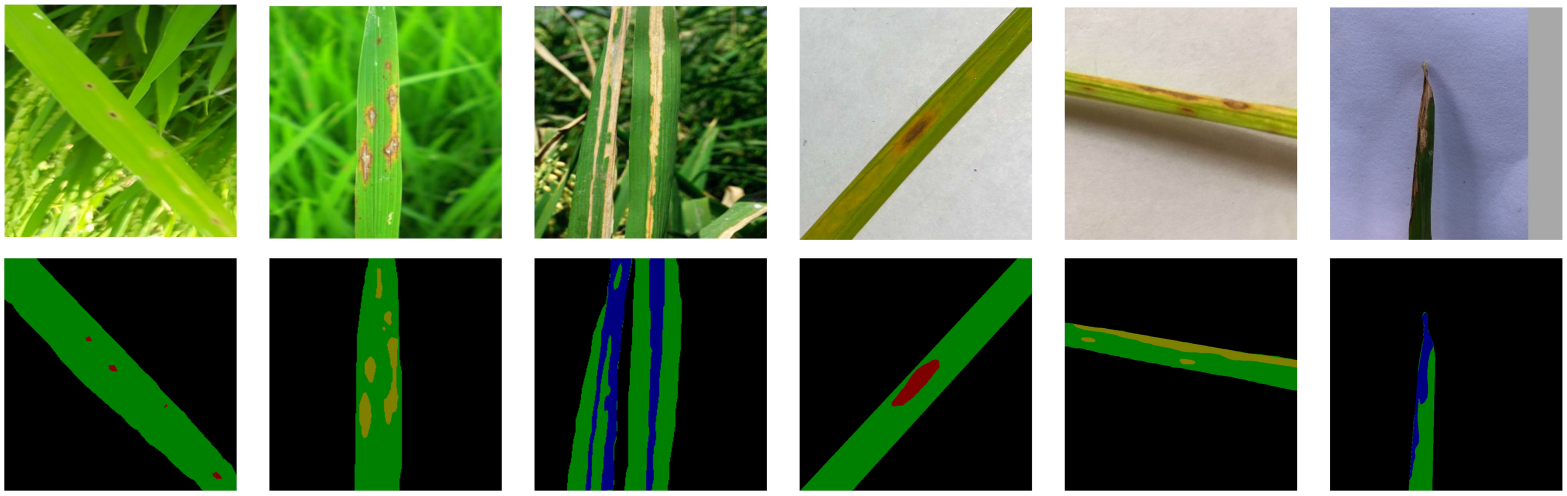
Original images and corresponding mapping mask images.

The validation set remained unaugmented to ensure objective performance evaluation. Grayscale mask labels were generated using Labelme, assigning five target categories: background (0), healthy leaves (1), brown spot (2), rice blast (3), and bacterial blight (4). **Figure 1** presents representative examples of images with different background complexities and their corresponding labeled masks. All data were converted into PASCAL VOC format before training.

### 2.2 DeepLabv3+ model

The DeepLab series of models are semantic segmentation networks developed by Google (Chen et al., 2014, 2017, 2018a, 2018b), with DeepLabv3+ representing the latest iteration. This model employs an encoder-decoder architecture. The encoder module consists of the Xception backbone network and the Atrous Spatial Pyramid Pooling (ASPP) module. The ASPP module contains a 1×1 convolutional layer, dilated convolutions with different dilation rates (6, 12, and 18), and a global average pooling layer. The encoder uses Xception for initial feature extraction, generating high-resolution low-level features containing edge and texture details, and low-resolution high-level features rich in semantic information. The obtained high-level features are aggregated across multiple scales through the ASPP module to generate the final high-level features. In the decoder module, low-level image features are upsampled 4× via bilinear interpolation and fused with high-level image features, followed by a 3×3 convolution to restore detailed features. Finally, another 4× upsampling is applied to recover spatial information and achieve pixel-level segmentation. While DeepLabv3+ extracts high-level and low-level image features through its encoder-decoder structure and leverages the ASPP concept to capture clearer object boundaries, its substantial computational cost, combined with the dense and small rice disease lesions and complex rice growth environments, poses new challenges for the model.

### 2.3 Rice leaf disease classification model based on MMPC-DeepLabv3+

**Figure 2:**
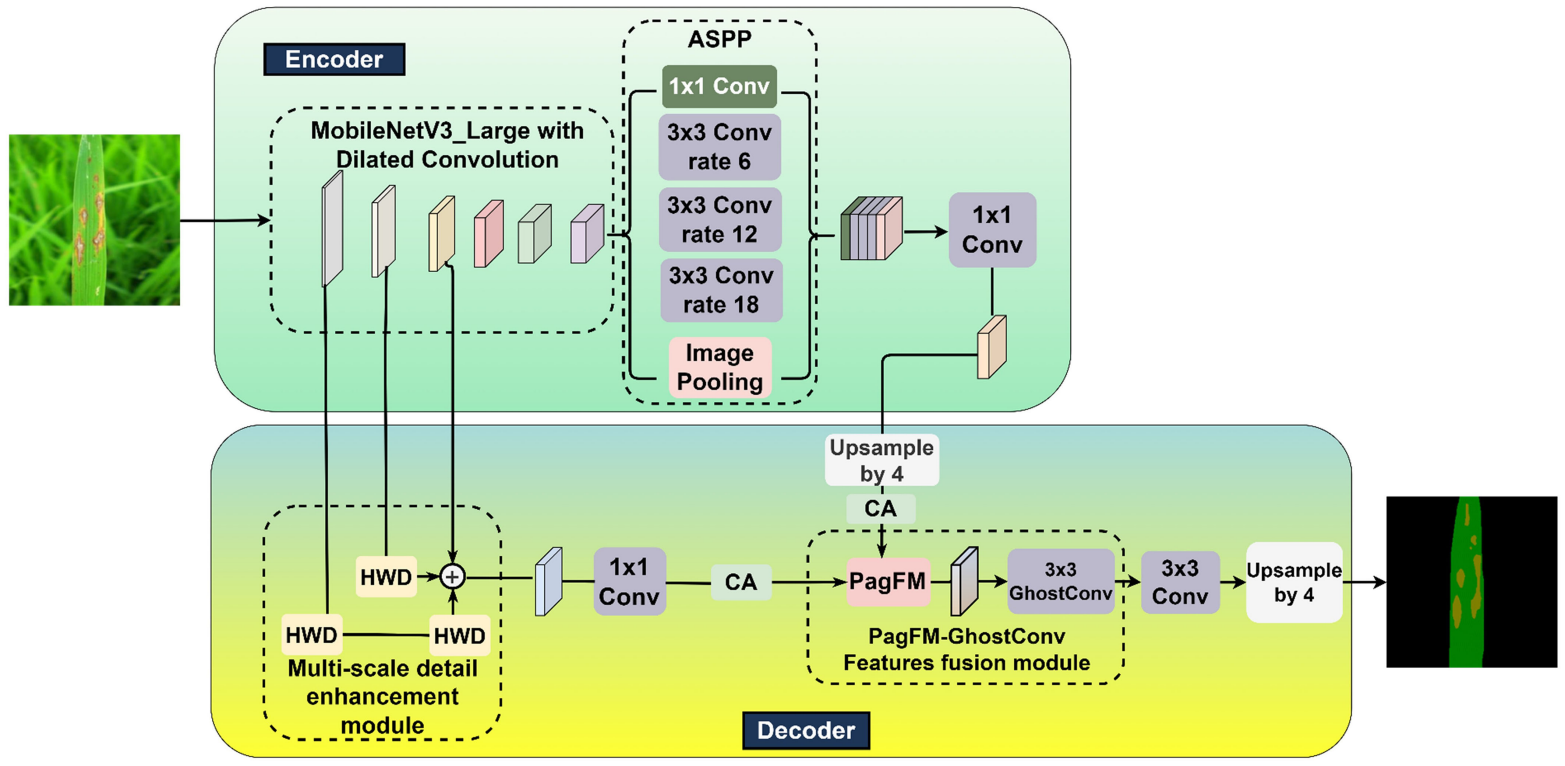
MMPC-DeepLabv3+ architecture diagram.

As delineated in **Figure 2**, a novel MV3L-MSDE-PGFF-CA-DeepLabv3+ (MMPC-DeepLabv3+) architecture was developed in this investigation for rice leaf lesion segmentation through strategic modifications of the DeepLabv3+ framework.

The principal modifications in the proposed MMPC-DeepLabv3+ architecture are reflected in its four core components, from which the name “MMPC” is derived:: (1) M – MobileNetV3-Large (MV3L): Replaces the conventional Xception backbone with a lightweight and efficient MV3L network, specifically designed to enhance semantic segmentation performance while significantly reducing computational complexity.; (2) M – Multi-Scale Detail Enhancement (MSDE): Introduces a detail enhancement module to improve edge detection and low-level texture extraction, thereby enabling more accurate lesion boundary delineation through richer hierarchical representations. (3) P – PagFM-GhostConv Feature Fusion (PGFF): Implements an optimized high-low feature fusion module that not only preserves critical semantic-spatial information but also reduces FLOPs and parameter count, promoting lightweight deployment. (4) C – Coordinate Attention (CA): Applies attention enhancement to both high- and low-level features prior to concatenation, strengthening the model’s focus on lesion regions under complex backgrounds and improving segmentation robustness.

These modular innovations collectively support the lightweight, accurate, and attention-enhanced segmentation capabilities of MMPC-DeepLabv3+, making it well-suited for field-level agricultural applications.

#### 2.3.1 MobileNetV3-large backbone network

MV3L (Howard et al., 2019), developed by Google, is a lightweight CNN network designed to achieve efficient and accurate visual recognition while minimizing computational resource usage, making it suitable for visual recognition tasks in field environments with limited computational resources. In MV3L, the Squeeze-and-Excitation (SE) attention mechanism is incorporated into select bottleneck (Bneck) blocks. The SE mechanism uses two fully connected layers to capture channel-wise dependencies and generate channel weights.

**Figure 3:**
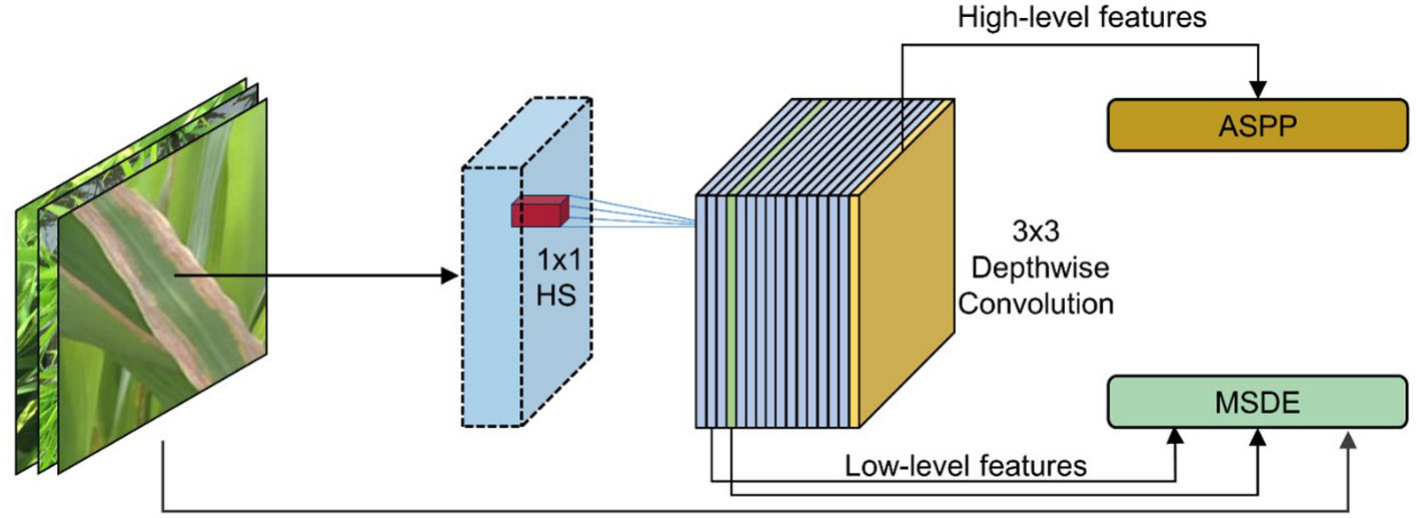
Optimized MV3L architecture.

In this study, the lightweight network MV3L was selected as the feature extraction network for DeepLabv3+, but adjustments were made to MV3L to accommodate the spatial information retention requirements of semantic segmentation tasks (**Figure 3**). Specifically, to preserve spatial information, the global average pooling layer and subsequent fully connected layers—originally designed for image classification tasks—were removed from the MV3L network, retaining only the spatial information downsampled by 32× through MV3L. However, the spatial information from 32× downsampled features remained insufficient for the requirements of DeepLabv3+. Consequently, the downsampling rate in MV3L was adjusted to 16× to address this limitation.

The specific operation was to set the stride of the 5×5 convolution in the Bneck structure (layer 14 in **Table 1**) within the original MV3L, which performs 32× downsampling, to 1. Secondly, to compensate for the receptive field, the dilation rate of the 5×5 convolution in the Bneck of the 14th layer was set to 2 to make up for the reduction of the receptive field caused by the decrease of the downsampling rate. Finally, the output from the 16th layer in the MV3L was directly utilized as high-level features for subsequent processing through the ASPP module.

Following the adjustments and optimizations applied to MV3L, the model not only retains the strong feature extraction capabilities inherent to the MV3L architecture but also achieves a reduction in Params and Flops. As shown in **Table 1**, the outputs of layer 4 and layer 16 are used as low-level and high-level features in DeepLabv3+ respectively. Additionally, the output of layer 2 will be utilized in the subsequent multi-scale detail enhancement module.

**Table 1:** MobileNetV3-Large feature extraction network configuration.

Number of layers	Shape	Form	Dilation rate	Output channels	SE	Activation function	Stride
1	512×512×3	conv2d	—	16	—	HS	2
2	256×256×16	Bneck	—	16	—	Relu	1
3	256×256×16	Bneck	64	24	—	Relu	2
4	128×128×24	Bneck	72	24	—	Relu	1
5	128×128×40	Bneck	72	40	✓	Relu	2
6	64×64×40	Bneck	120	40	✓	Relu	1
7	64×64×40	Bneck	120	40	✓	Relu	1
8	64×64×40	Bneck	240	80	—	HS	2
9	32×32×80	Bneck	200	80	—	HS	1
10	32×32×80	Bneck	184	80	—	HS	1
11	32×32×80	Bneck	184	80	—	HS	1
12	32×32×80	Bneck	480	112	✓	HS	1
13	32×32×112	Bneck	672	112	✓	HS	1
14	32×32×112	Bneck	672	160	✓	HS	1
15	32×32×160	Bneck	960	160	✓	HS	1
16	32×32×160	Bneck	960	160	✓	HS	1

The "✓" denotes whether the Squeeze-and-Excitation (SE) attention module is integrated into each specified layer of the MobileNetV3-Large feature extraction network.

#### 2.3.2 Multi-scale detail enhancement module

**Figure 4:**
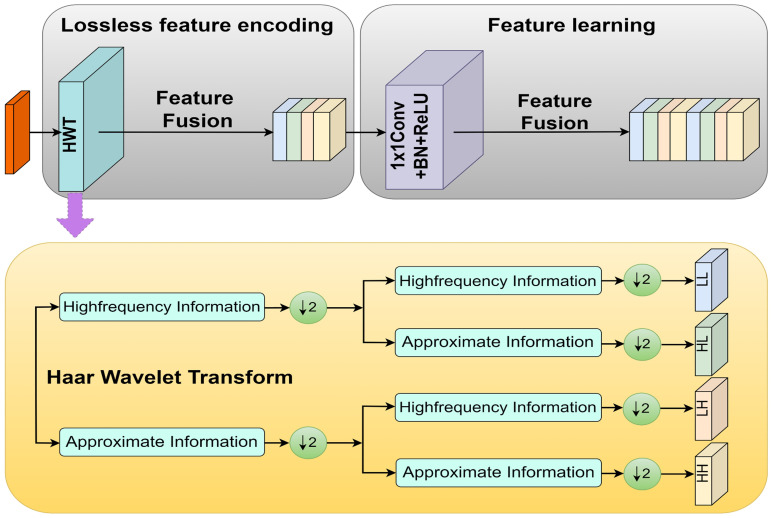
HWD module.

To improve the capability of the model in capturing fine-grained details, Haar Wavelet Downsampling (HWD) technology was incorporated. As shown in **Figure 4**, the HWD module can reduce image resolution while effectively preserving detailed information in three directions: horizontal, vertical, and diagonal (Xu et al., 2023a). For input feature maps, the process involved three steps: (1) applying Haar wavelet transform for 2× downsampling, generating four subbands: Low-Low (LL), Low-High (LH), High-Low (HL), and High-High (HH); (2) stacking these subbands along the channel dimension for feature fusion; (3) adjusting the number of channels via 1×1 convolution to achieve efficient feature integration.

**Figure 5:**
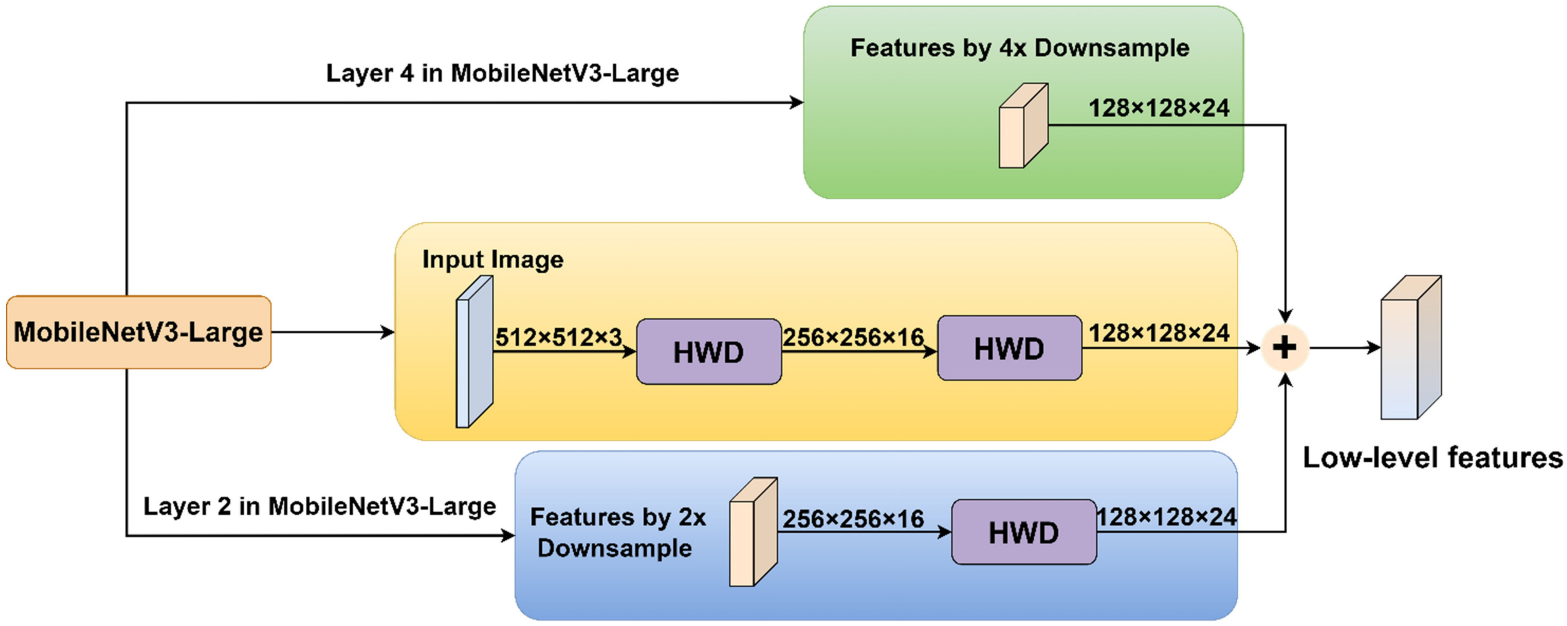
MSDE module.

Based on the information capture and processing capabilities of the HWD module, this study introduced it into the model to propose the MSDE module (**Figure 5**). The module consisted of three parallel branches that extracted and integrated detailed information from different levels: Branch 1: Applied the HWD module twice to achieve 4× downsampling of the input image, extracting fine details directly from the original input. Branch 2: First downsampled the input 2× through the feature extraction network, then applied HWD for an additional 2× downsampling (total 4×), aiming to capture detailed information from the medium-depth networks. Branch 3: Directly utilized the 4× downsampled features from the feature extraction network to obtain preliminarily abstracted low-level features.

The final step involved element-wise addition of the three branch outputs to generate a fused feature map containing multi-scale detail information. This fusion strategy enriched low-level features with edge, corner, and texture details, significantly improving the performance of the model in segmenting lesion boundaries and small targets.

In implementation details, the input image of size 512×512×3 undergoes two successive HWD transformations: first reducing to 256×256×16 and then further downsampling to 128×128×24. Simultaneously, the 2× downsampled 256×256×16 feature map from layer 2 in the MV3L architecture (**Table 1**) is processed through the HWD module, yielding a 128×128×24 feature map. These three identically dimensioned feature maps are combined through element-wise addition to generate the final low-level features.

#### 2.3.3 Introduction of the coordinate attention mechanism

To further enhance the feature representation capabilities of the model, a CA mechanism (Hou et al., 2021) was applied to both the high-level features from the ASPP module and the low-level features extracted by the multi-scale detail enhancement module. This attention mechanism helped the model better understand the spatial distribution of lesions across the entire leaf surface, improving its robustness in complex segmentation scenarios.

**Figure 6:**
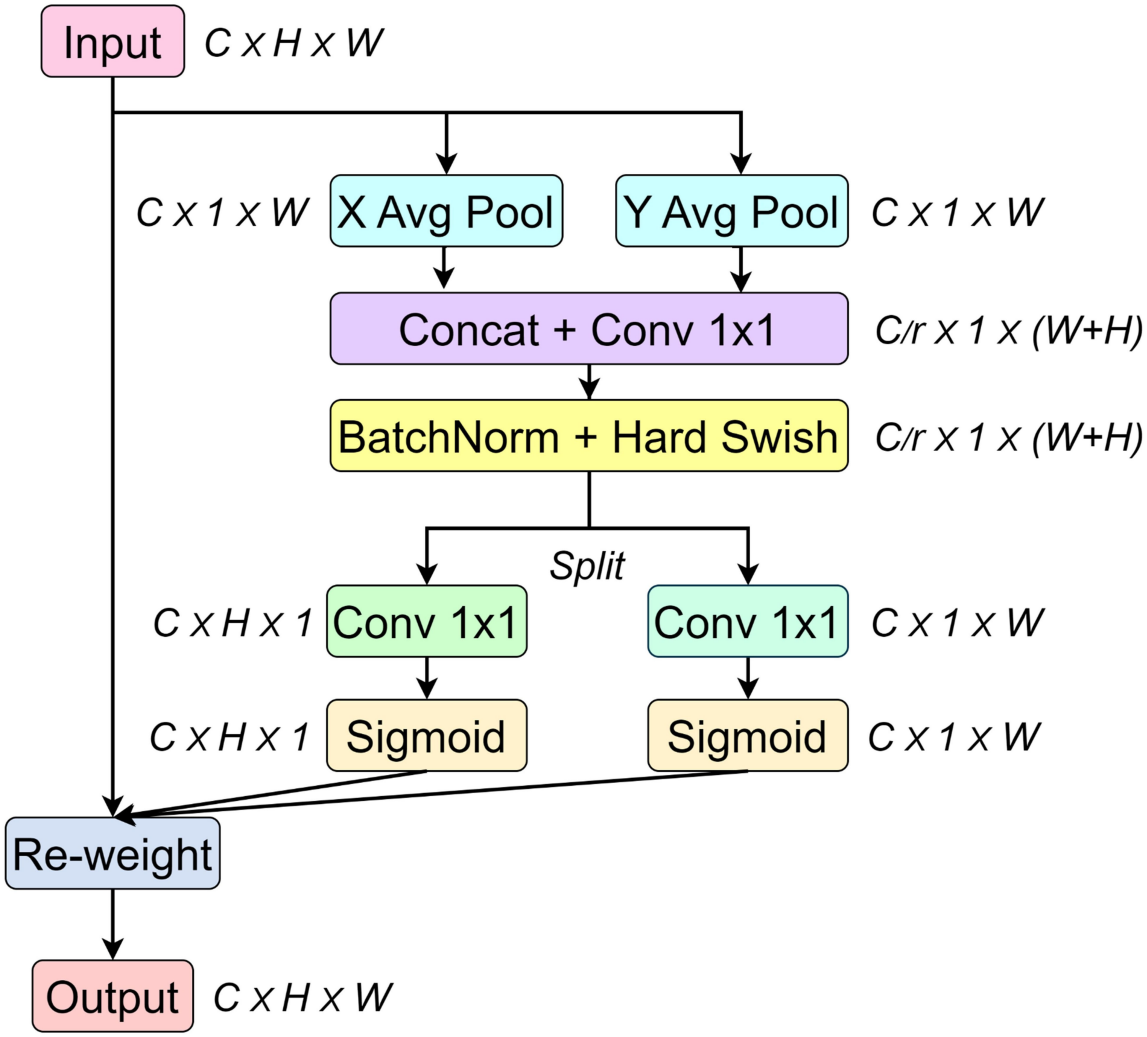
CA mechanism.

The CA mechanism introduces explicit coordinate information based on channel attention to enhance the ability of the model to understand the spatial distribution information in the input features. As shown in **Figure 6**, by obtaining the spatial information about the width and height of the image to enhance feature representation, the input feature map is decomposed into horizontal and vertical directions through global average pooling, and two global context features are generated.

Subsequently, the two global context features are concatenated along the channel axis. By applying a 1×1 convolution, the number of channels is adjusted to C/r (where C is the number of channels, and in this paper, the hyperparameter. is set to 16, meaning the number of intermediate channels is 1/16 of the number of input channels). Then, the stacked feature vectors are separated into the height and width directions. Next, the number of channels is adjusted back to the number of input channels using a 1×1 convolution. Through the sigmoid function, the feature values are mapped to the range from 0 to 1, generating the attention maps in the height and width directions.

Finally, the original input and the attention maps in the H and W directions are multiplied element by element to complete the feature recalibration, which highlights the important features and suppresses the unimportant regions.

#### 2.3.4 Lightweight high-low feature fusion module

**Figure 7:**
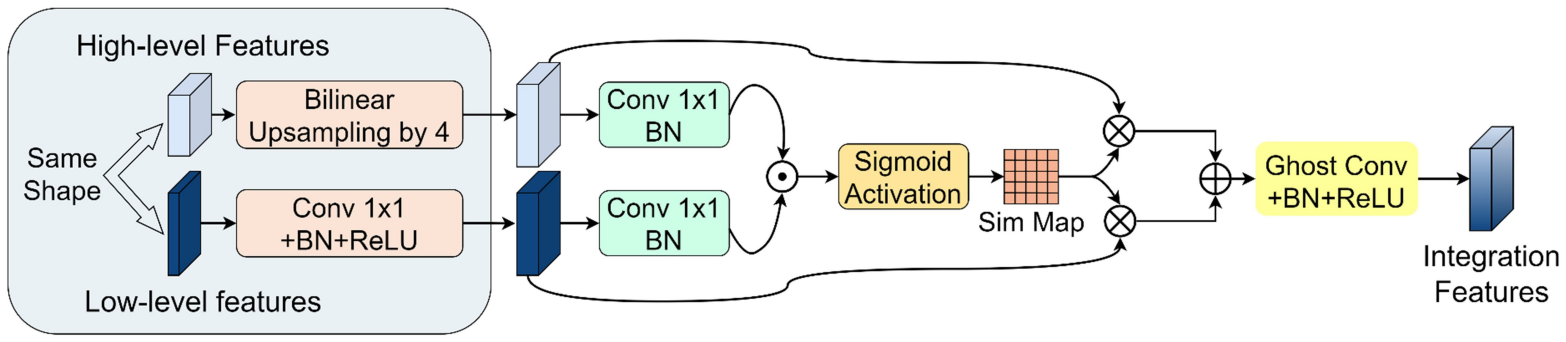
PGFF module.

A novel PGFF module was proposed for this study, inspired by the Pixel-attention-guided Fusion Module (PagFM) architecture described in (Xu et al., 2023b). Specifically, the module integrates high-level semantic features and low-level spatial features through a two-stage processing pipeline. As illustrated in **Figure 7**, the shapes of multi-scale feature maps were first aligned. To reduce parameters, a 1×1 convolution was applied to halve the number of channels. Then the similarity derived from sigmoid mapping was multiplied element-wise. This result was employed as weighting coefficients for feature interaction. The preliminary fused features were subsequently synthesized through weighted summation, followed by final feature generation via 3×3 Ghost convolution operations. Notably, the PagFM component employs dimensionality reduction via 1×1 convolution to mitigate computational overhead during feature interaction. **Table 2** presents a comparative analysis demonstrating that the proposed module reduced Params and Flops compared to the original fusion methods.

**Table 2:** Comparison of parameter and computational cost between two feature fusion methods.

Feature fusion method	Params/M	Flops/G
Original model stack fusion	1.293	42.39
PagFM-Ghostconv fusion	0.14	4.702

#### 2.3.5 Improvement of the loss function

In preliminary experiments, the segmentation model trained using only the Dice loss function failed to achieve satisfactory results due to the class imbalance in the rice leaf image dataset (**Table 3**), where healthy tissues and background regions occupied the majority of pixels, while disease lesions accounted for only a small proportion. The resulting masks exhibited severe under-segmentation, especially in small or indistinct lesion regions. This highlighted the necessity of guiding the attention of the model toward hard-to-classify and underrepresented categories. To address this issue, Focal Loss (Lin et al., 2017) was adopted Specifically, as formulated in **Equation 1**, a modulating factor ${\left(1-p_{t}\right)}^{\gamma }$ was added by introducing the method modifies the cross-entropy loss function, where $p_{t}$ denotes the predicted probability for each class and γ serves as a hyperparameter controlling the degree of focus. This formulation effectively down-weights the loss contribution from easy-to-classify samples, thereby shifting the attention of the model toward hard-to-classified samples. The mathematical expression of Focal Loss is defined as follows:

**Table 3:** Pixel quantity and category weight of each class.

Categories	Label values	Number of Pixels	Weights
Background	0	515499053	0.0174
Healthy Leaf	1	85752875	0.0669
Brown Spot	2	2327595	1
Rice Blast	3	4321519	0.6287
Bacterial Blight	4	6882958	0.4435


(1) $$Focal\;Loss\text{ = }-\alpha_{t}{\left(1-p_{t}\right)}^{\gamma }\log(p_{t})$$

Where, $\alpha_{t}$ enotes a class-specific weight parameter designed to treat the imbalance of categories.

Category weight parameters $\alpha_{t}$ were computed based on pixel distribution statistics across dataset categories, with values presented in the final column of **Table 3**. Although Focal Loss partially mitigates class imbalance, as a cross-entropy loss function, it chiefly focuses on pixel-wise classification accuracy, limiting its ability to resolve complex lesion morphologies. To address this limitation, Dice Loss (Milletari et al., 2016)was integrated into the framework. This loss function explicitly targets segmentation accuracy by comparing the similarity between model-predicted segmentation results and truth labels, as defined in **Equation 2**:


(2) $$Dice\;Loss\text{ = }1-\frac{2\times \left|X\cap Y\right|+smooth}{\left|X\right|+\left|Y\right|+smooth}$$

Where, X and Y represent the predicted and true labels, respectively; smooth is a non-zero value to avoid a zero denominator.

The final composite loss function in this study is formulated in **Equation 3**:


(3) Loss=Focal Loss+Dive Loss

This Loss function strategy, which combines pixel-level Focal Loss and region-level Dice Loss, not only improves the model ability to identify and process difficult-to-classify pixels, but also ensures accurate segmentation of the lesion area.

### 2.4 Model training and testing

#### 2.4.1 Model training environment

In this study, based on the constructed model, tasks such as model training and evaluation were carried out to verify the effectiveness of the improved model proposed herein. The configurations of the software and hardware environments for the experiments are presented in **Table 4**.

**Table 4:** Experimental environment configuration.

Software and hardware	Version
CPU	Gen Intel(R) Core(TM) i7-13700K
GPU	NVIDIA GeForce RTX 3090(24GB)
Operating system	Windows 10
Anaconda	4.3.0
CUDA	11.0
Cudnn	8.0.5
Python	3.8.18
Pytorch	1.7.1

PyTorch played a pivotal role in the subsequent segmentation tasks. Through the Application Programming Interfaces (APIs) it provided, the model architecture could be rapidly defined and adjusted, enabling fine control over the model. Notably, PyTorch supported GPU acceleration, and when used in conjunction with CUDA and CuDNN, it significantly shortened the experimental cycle. To avoid package conflicts, Anaconda was employed to construct a Python virtual environment and manage various package dependencies.

#### 2.4.2 Test setups

Transfer learning (Yosinski et al., 2014) allows researchers to apply existing knowledge to new tasks by utilizing pre-trained weights obtained from large-scale datasets, thereby enhancing the learning efficiency and performance of the new tasks. Therefore, in this paper, the model training was conducted based on transfer learning. Firstly, the weights of the feature extraction network were initialized using the weights of the pre-trained model. Secondly, during the initial stage of training, the feature extraction network was frozen, and only the custom layers were trained. Finally, the feature extraction network was unfrozen, allowing it to update its weights, and the network was fine-tuned. In this paper, the total number of training iterations was set to 300 epochs, among which 50 epochs were for the frozen training and 250 epochs were for the unfrozen training.

During the frozen stage, the batch size was set to 16 and the initial learning rate was set to 5×10^-4^. During the unfrozen stage, considering the increased memory load due to backpropagation through the backbone, the batch size was reduced to 8. A cosine annealing learning rate scheduler was employed throughout to gradually decay the learning rate, with the minimum learning rate set to 5×10^-6^. The optimizer used was Adam, with a momentum coefficient of 0.9. The input images were resized to a fixed resolution of 512×512 and processed as standard 3-channel RGB images. The loss function adopted was a hybrid combination of Focal Loss and Dice Loss, which balances pixel-level focus with region-level consistency to handle class imbalance and improve lesion segmentation accuracy.

To ensure the reliability of model performance and reduce the impact of random fluctuations during training, the model weights were saved every 5 epochs throughout the 300 training epochs. After training, the model checkpoint with the highest MIoU on the training set was selected, and its performance was evaluated on the validation set. This procedure was uniformly applied to all models in the comparison experiments, ensuring consistency and fairness across evaluations.

#### 2.4.3 Model evaluation metrics

In this study, to comprehensively evaluate the segmentation accuracy of the model, the MIoU and the MPA were adopted as the primary evaluation metrics. These two metrics assess the segmentation performance of the model from both the overall and pixel levels respectively. Furthermore, the Params and Flops are adopted as comprehensive metrics to evaluate performance and computational efficiency (Xie et al., 2024). In this study, Params is calculated by aggregating trainable parameters across all architectural layers, reflecting model complexity and storage requirements. Flops quantifies the total floating-point operations required during a single forward pass, serving as a critical indicator of computational load. The formulas for these metrics are given in **Equations 4**–**11**:


(4) $$MPA=\frac{1}{n}\sum_{i=1}^{n}\frac{p_{ii}}{\sum_{j=1}^{n}p_{ij}}$$

(5) $$MIoU=\frac{1}{n}\sum_{i=1}^{n}\frac{m_{ii}}{\sum_{j=1}^{n}m_{ij}+\sum_{j=1}^{n}m_{ji}-m_{ii}}$$

(6) $$params_{conv}=(kernel_{size}\times C_{in}+1)\times C_{out}$$

(7) $$params_{fcl}=(f_{in}+1)\times f_{out}$$

(8) $$params_{BN}=2\times C_{in}$$

(9) $$Params=params_{conv}+params_{fcl}+params_{BN}$$

(10) $$Precision=\frac{p_{ii}}{p_{ii}+FP}$$

(11) $$mPrecision=\frac{1}{n}\sum_{i=1}^{n}Precision$$

where, $p_{ii}$ represents the number of pixels correctly classified into category i; $p_{ij}$ is the number of pixels that actually belong to category i but are misclassified into category j; and n is the total number of categories. $m_{ii}$ is the number of pixels that are correctly predicted, that is, pixels belonging to class i are predicted as class i, while $m_{ij}$ is the number of pixels that are wrongly predicted, meaning pixels belonging to class i are predicted as class j. $params_{conv}$ is the number of parameters in the convolutional layer; $params_{fcl}$ is the number of parameters in the fully connected layer; $params_{BN}$ is the number of parameters in the batch normalization layer; $kernel_{size}$ is the size of the convolutional kernel; $C_{in}$ is the number of input channels; $C_{out}$ is the number of output channels; $f_{in}$ is the number of input channels of the fully connected layer; $f_{out}$ is the number of output channels of the fully connected layer; and Params is the total number of parameters; FP is the number of pixels from other classes j that were misclassified as class i.

## 3 Results and analysis

### 3.1 Baseline model determination experiments

**Figure 8:**
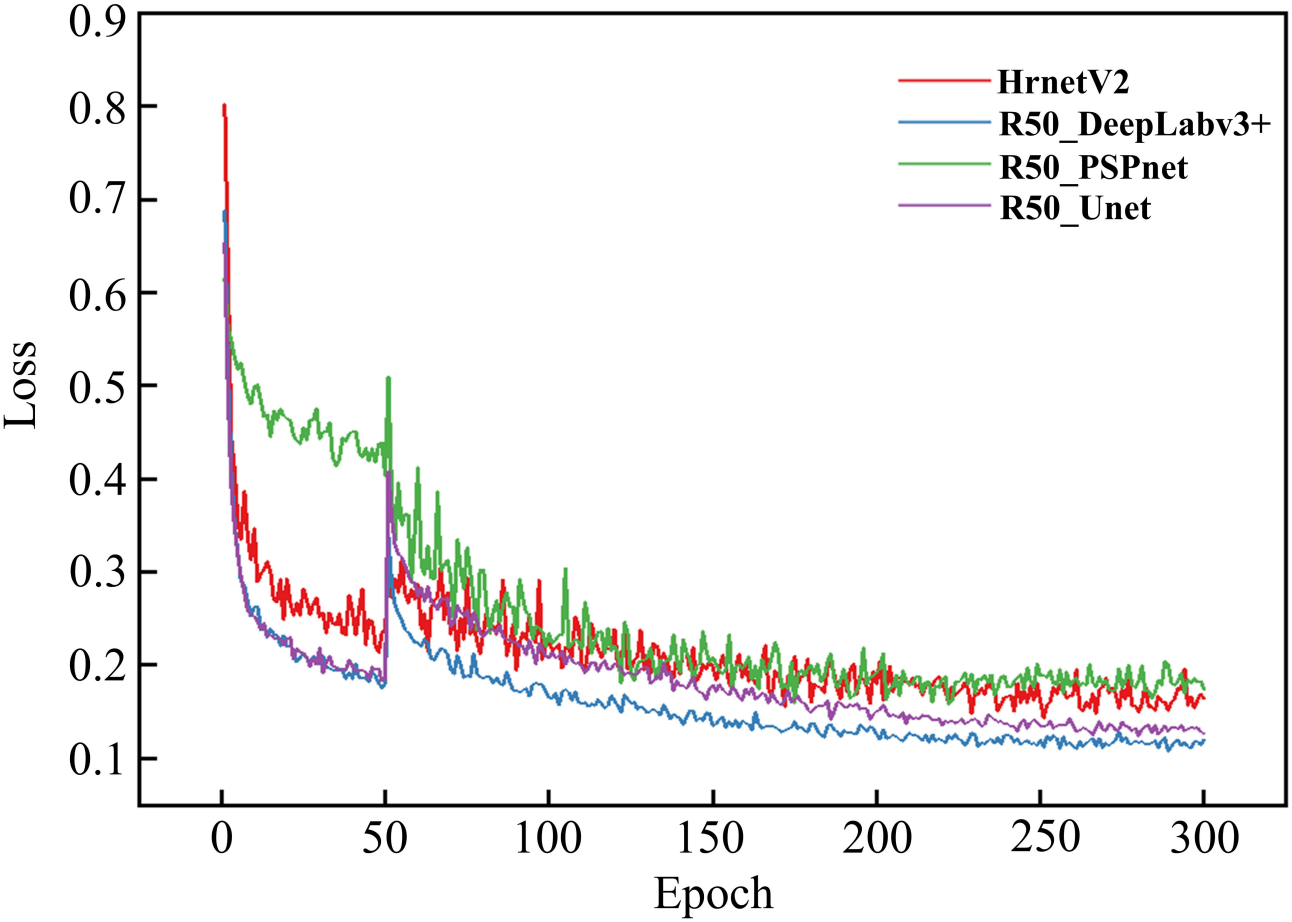
Training loss evolution across different models.

To validate the reliability and accuracy of DeepLabv3+ as the baseline model in this study, four models, namely DeepLabv3+, Unet (Ronneberger et al., 2015), PSPNet (Zhao et al., 2017), and HRNetV2 (Wang et al., 2021), were systematically evaluated through comparative training and testing on identical datasets. During the training process, the feasibility of the models was evaluated by examining the changes in model loss and the segmentation evaluation metrics of different disease lesions in the validation set. **Figure 8** shows the changes in the loss ratios of the four models on the training set, and **Table 5** presents the results of the metric evaluation.

**Table 5:** Performance of different models on the validation set.

Model	Backbone	MIoU/%	MPA/%	Flops/G	Params/M
**DeepLabv3+**	**ResNet50**	**79.8**	**89.63**	**113.27**	**27.822**
Unet	ResNet50	79.34	88.96	184.2	43.933
PSPNet	ResNet50	75.24	87.41	118.43	46.708
HRNetV2	Hrnet_w32	78.26	88.28	90.972	29.540

Deeplabv3+ represents the baseline model used in this study after balancing the trade-off between accuracy and computational cost. ResNet50 indicates that the backbone used in Deeplabv3+ is ResNet50. 79.8 value represents the best performance of the MIou metrics. 89.63 value represents the best performance of the MPA metrics. 113.27 value represents the second-lowest Flops among all compared models. 27.822 value represents the smallest Params among all compared models.

As illustrated in **Figure 8**, all models demonstrated rapid loss decline during initial training phases, primarily attributed to the introduction of pre-trained weight enabling efficient feature extraction from agricultural imagery. Unfrozen training commenced at epoch 51, during which weights in the feature extraction network were updated. Since the parameters had not yet fully adapted to the dataset at this time, there was a sudden jump in the loss values of the four models.

Notably, as the training progressed, the losses of all four models gradually decreased and eventually stabilized. This indicates that all four architectures are capable of effectively distinguishing disease types and achieving the segmentation of disease lesions. It is worth noting that the R50-DeepLabv3+ model reached a relatively low convergence value for its loss. To some extent, this also demonstrates a high degree of compatibility between this architecture and the task of disease lesion segmentation.

As shown in **Table 5**, all four models performed quite well in the evaluation of the two accuracy indicators, MIoU and MPA. The MIoU values exceeded 75%, and the MPA values were around 88%. The evaluation based on these two indicators once again verified the feasibility of using encoder-decoder structures for segmenting rice disease spots. DeepLabv3+ had MIoU and MPA values of 79.8% and 89.63% respectively, which were the highest among the four models. In the evaluation of model efficiency and complexity, the Params of DeepLabv3+ was 27.822 M, the lowest among the four models. HRNetV2 had the least amount of Flops, which was 90.972 G, and followed by DeepLabv3+ with 113.27 G Flops. However, both the MIoU and MPA of DeepLabv3+ were significantly higher than those of HRNetV2. After comprehensive consideration, DeepLabv3+ was selected as the baseline model for the segmentation of rice disease spots in this paper.

### 3.2 Comparison experiment of different backbone

**Figure 9:**
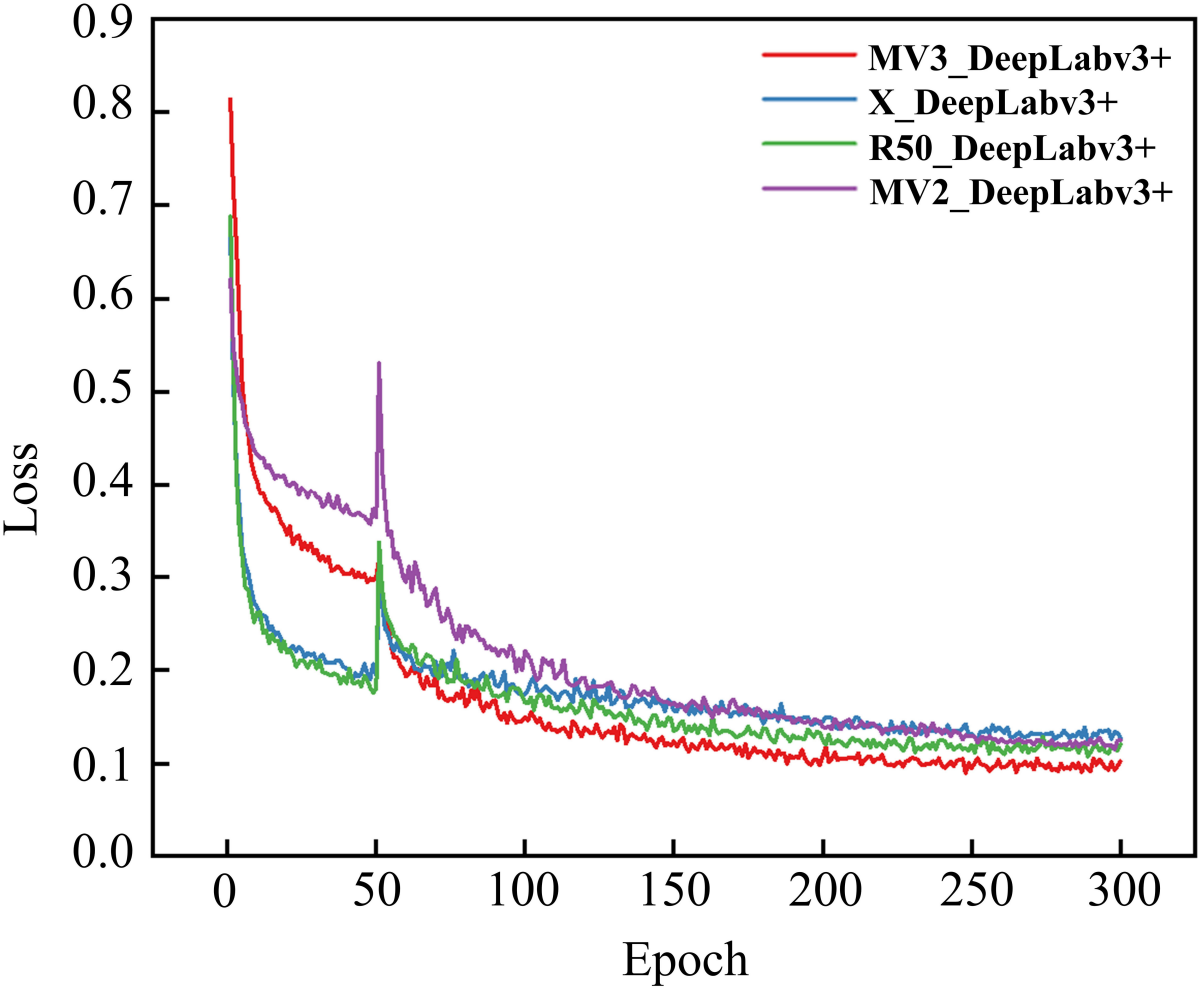
Variation in training loss of DeepLabv3+ models utilizing various backbones.

To systematically evaluate the impact of different backbone architectures on DeepLabv3+ performance for rice lesion segmentation, four feature extraction networks, namely Resnet50, Xception, MobilenetV3_large, and MobilenetV2, were comparatively analyzed under the same experimental framework. These variants were designated as R50_DeepLabv3+, X_DeepLabv3+, MV3L_DeepLabv3+, and MV2_DeepLabv3+, respectively. **Figure 9** presents the training loss progression curves, while **Table 6** presents the comparison results of different feature extraction networks in various indicators.

**Table 6:** Comparison of experimental results using different backbones.

Model	Backbone	MIoU/%	MPA/%	Flops/G	Params/M
R50_DeepLabv3+	ResNet50	79.8	89.63	113.27	27.822
X_DeepLabv3+	Xception	79.34	88.96	141.21	42.181
**MV3L_DeepLabv3+**	**MV3L**	**80.08**	**89.66**	**47.024**	**4.651**
MV2_DeepLabv3+	MobilenetV2	78.58	88.3	48.883	3.856

MV3L_DeepLabv3+ denotes the proposed enhanced model, which improves upon the standard DeepLabv3+ architecture with MV3L as the backbone. MV3L represents the optimal backbone for DeepLabv3+ in terms of performance. 80.08 value represents the best performance of the MIou metrics. 89.66 value represents the best performance of the MPA metrics. 47.024 value represents the smallest Flops among all compared models. 4.651 value represents the second-lowest Params among all compared models.

As shown in **Figure 9**, the training losses of all models showed a downward trend as the training progress, indicating that the model performance was gradually improving. Specifically, X_DeepLabv3+ and MV3L_DeepLabv3+ exhibited faster initial convergence rates, and R50_DeepLabv3+ and MV2_DeepLabv3+ displayed slower loss attenuation rates. When approaching the 200th epoch after the unfrozen training, the loss values of all models gradually decreased and tended to be stable, indicating that the models had converged. Among them, MV3L_Deeplabv3+ achieved the lowest loss value, demonstrating that this feature extraction network has a better performance in the DeepLabv3+ model. In contrast, the loss values of X_DeepLabv3+, R50_DeepLabv3+, and MV2_DeepLabv3+ were slightly higher, but they still showed a good convergence trend.

As shown in **Table 6**, different backbones have varying impacts on the performance of the model, especially having a relatively large influence on the two indicators of the Params and Flops. In terms of segmentation accuracy, when MV3L is used as the backbone, the segmentation accuracy is the highest, with the MIoU being 80.08%. When MobileNetV2 serves as the backbone, the MIoU is the lowest, reaching 78.58%. Regarding model efficiency, when Resnet50 and Xception are employed as backbones, the computational cost is relatively high and the lightweight MobileNetV2 and MV3L have the lowest computational costs, showing a significant reduction in the Params, with the maximum reduction rate being 90.7%. Among them, the Flops of MV3L and MobileNetV2 are 47.024 G and 48.883 G, respectively, and the Params are 4.651 M and 3.856 M, respectively.

Typically, Params and Flops demonstrate a positive correlation under identical input size. However, in this study, an inverse relationship was observed when MobileNetV2 was integrated with DeepLabv3+. This phenomenon stems from discrepancies in the number of channels of the last layer of feature maps when the two backbones are transmitted to the ASPP.

Considering both segmentation accuracy and computational efficiency, MV3L as the backbone achieved the highest accuracy among the four evaluated architectures. Although Params of the MV3L was 0.785 M higher than MobileNetV2, it demonstrated superior performance with a 1.5% increase in MIoU and a 1.36% improvement in MPA. Collectively, these results indicate that MV3L strikes a better balance between segmentation accuracy and model complexity when integrated into the DeepLabv3+ framework.

### 3.3 Comparative experiments on different attention mechanisms

In this study, based on the MV3L feature extraction network, a multi-level low-feature fusion module was designed, and a lightweight feature fusion method PagFM-GhostConv was adopted. Four lightweight attention mechanisms, namely CA, CBAM (Woo et al., 2018), CCA (Deng et al., 2022), and ECA, were respectively added, before feature fusion. As shown in **Table 7**, adding lightweight attention mechanisms did not impose significant computational burdens on the model (within 2.2%). Among them, the CA attention mechanism achieved the greatest improvement in model performance, with a 1.21% increase in MIoU and a 0.7% increase in MPA. The ECA attention mechanism ranked second in performance improvement, with a 0.42% increase in MIoU and a 0.25% increase in MPA. The CBAM and CCA attention mechanisms showed limited improvements in model performance. These results verified the effectiveness of adding the CA attention mechanism.

**Table 7:** Comparative experiments on different attention mechanisms.

Position	Attention mechanism	MIoU /%	MPA /%	Flops /G	Params /M
	\	80.02	89.29	9.657	3.549
Before feature fusion	**CA**	**81.23**	**89.79**	**9.695**	**3.556**
	CBAM	80.19	89.78	9.68	3.558
	ECA	80.44	89.54	9.674	3.549
	CCA	80.14	89.41	15.076	3.632

CA represents the best-performing among the four attention mechanisms. 81.23 value represents the best performance of the MIou metrics. 89.79 value represents the best performance of the MPA metrics. 9.695 represents the FLOPS value of the selected attention mechanism. 3.556 represents the value of the Params for the selected attention mechanism.

### 3.4 Results and analysis of ablation experiment

To validate the individual contributions of the key architectural enhancements in MMPC-DeepLabv3+, a series of ablation experiments were conducted on the MV3L-based DeepLabv3+ framework. These experiments systematically assessed the impact of three critical modules: the MSDE module, the PGFF approach, and the CA attention mechanism. **Table 8** presents the detailed experimental results, highlighting the incremental contributions of each modification.

**Table 8:** Ablation experiment results.

Back bone	Experimental groups	MSDE	PGFF	CA	MIoU /%	MPA /%	Flops /G	Params /M
	Comparison group	×	×	×	80.08	89.66	47.024	4.651
MV3L	Group 1	✓	×	×	81.89	90.28	47.345	4.701
	Group 2	✓	✓	×	80.02	89.29	9.657	3.549
	Group 3	✓	✓	✓	81.23	89.79	9.695	3.556

"√" indicates adding a module into the the Deeplabv3+ model with MV3L as the Backbone.

"×" indicates the modules that have not yet been incorporated into the model.

In the first group of experiments, the proposed MSDE module was introduced to replace the conventional 4× downsampled low-level feature stream in DeepLabv3+. The motivation for this modification stems from the difficulty in accurately capturing small lesion regions and fine-grained boundaries using standard low-level features. By incorporating Haar wavelet decomposition, the MSDE module enables more expressive feature encoding of both primary and directional texture details. As a result, this enhancement achieved a 1.81% improvement in MIoU and a 0.62% gain in MPA, without significantly increasing the computational load. This demonstrates the effectiveness of MSDE in boosting boundary delineation and improving segmentation accuracy for subtle lesion areas.

In the second group of experiments, the MSDE-enhanced model was further optimized by integrating the PGFF module, which replaces the original DeepLabv3+ feature fusion strategy with a lightweight yet semantically adaptive approach. Unlike the conventional 4× upsampling and channel concatenation followed by two standard convolutions—which impose heavy computational burdens and struggle with semantic gaps between low-level and high-level features—PGFF first applies PagFM to reduce feature dimensionality and establish weighted semantic correspondence, then employs Ghost convolution for efficient refinement. This design achieves a 79.6% reduction in FLOPs and a 24.5% decrease in Params compared to the first group. While accuracy dropped slightly (MIoU −1.87%, MPA −0.99%), this trade-off was anticipated given the substantial efficiency gains.

Importantly, this design choice was made deliberately to enhance the suitability of the model for real-world deployment in resource-constrained agricultural environments, such as mobile phones, tablets, or embedded systems. In these scenarios, processing power and memory are often limited, and reducing model complexity is essential for ensuring smooth operation and real-time inference. Despite the slight reduction in accuracy, the model still maintained strong segmentation performance, indicating that the PGFF module effectively balances accuracy and efficiency. This validates the practical value of the optimization and supports its adoption in precision agriculture applications.

In the third group of experiments, the CA attention mechanism was introduced prior to feature fusion, enhancing both the high-level and low-level representations before being processed by PGFF. The motivation here is to further improve the model’s capacity to focus on salient lesion regions and suppress background noise under complex field conditions. This modification yielded a 1.21% improvement in MIoU and a 0.70% gain in MPA compared to the PGFF-only configuration, confirming the complementary effect of attention enhancement on lightweight fusion.

Finally, compared to the control group using MV3L_DeepLabv3+ as the feature extraction network, MMPC-DeepLabv3+ architecture achieved a 1.15% improvement in MIoU and a 0.13% increase in MPA while a 79.38% decrease in Flops, and a 23.5% reduction in Params. This optimization represents a significant advance in balancing segmentation performance and computational efficiency.

### 3.5 Performance comparison of different models

#### 3.5.1 Overall model performance comparison

**Figure 10:**
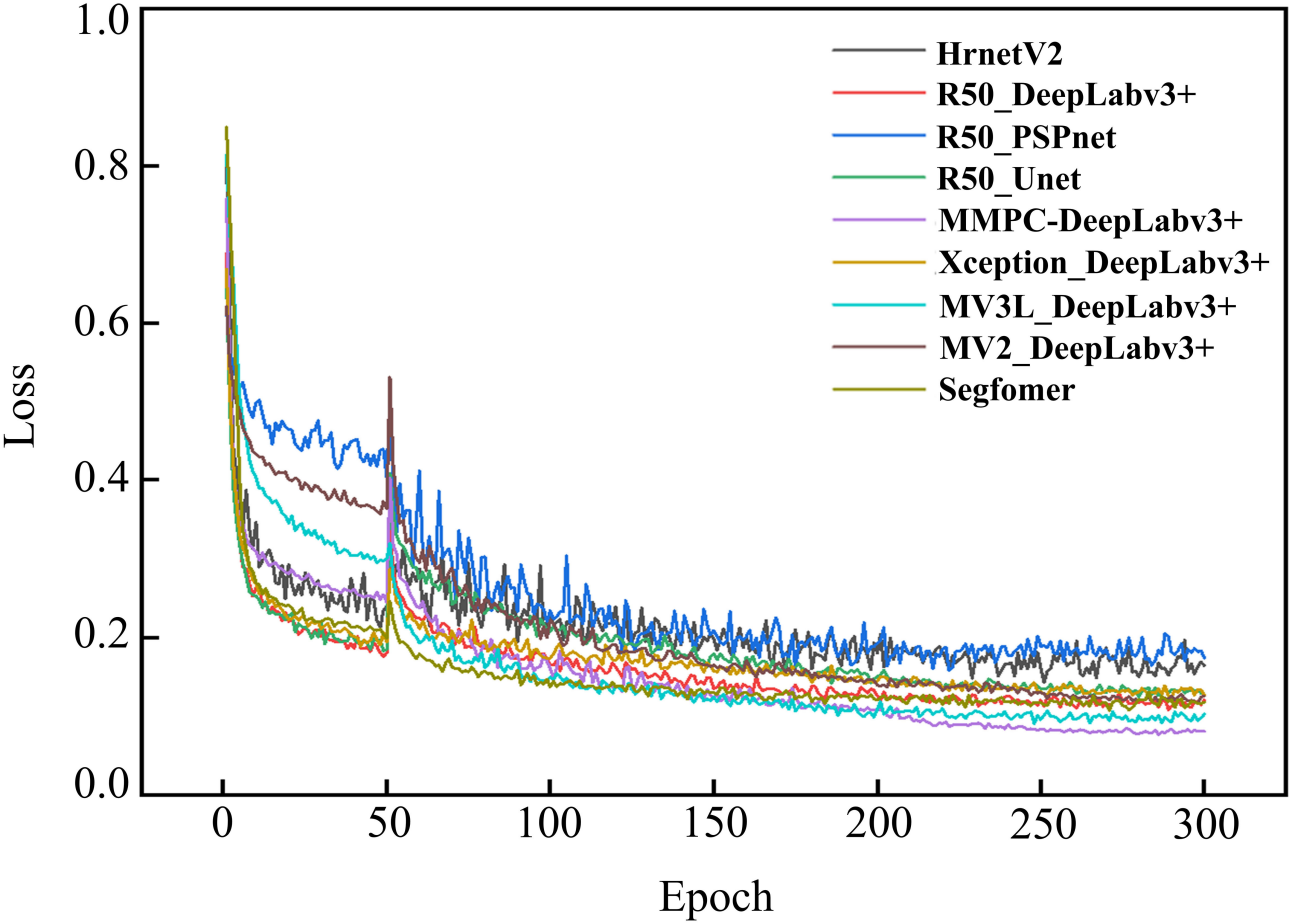
Training loss evolution across different models.

To evaluate the effectiveness of MMPC-DeepLabv3+ in rice lesion segmentation, longitudinal comparisons were conducted against mainstream models of rice lesion segmentation including Unet, PSPNet, HRNetV2, DeepLabv3+ variants with different feature extraction networks, and Segformer (Xie et al., 2021). **Figure 10** illustrates training loss evolution across different models. During the initial 50 epochs of frozen training, the loss curves of all models decreased as the number of epochs increased and gradually stabilized. During the subsequent 250 epochs of unfrozen training, the loss values of each model showed a downward trend as the training cycles increased and eventually stabilized, indicating that all models had reached a converged state. Special for the loss of the MMPC-DeepLabv3+ model, which started to stabilize after the 150th epoch of unfrozen training. As the training cycles continued to increase, the decrease in the loss value of the MMPC-DeepLabv3+ model was smooth, without significant fluctuations. At the end of the training, the loss value of the MMPC-DeepLabv3+ model was the lowest among all the compared models. The relatively low training loss value at convergence reflects its excellent fitting ability to the training data. This low loss value further validates the effectiveness of the improvements made in this study.

To evaluate the effectiveness of the proposed MV3L backbone in balancing spatial resolution and semantic abstraction, we conducted a comparative experiment by integrating different backbones into the DeepLabv3+ framework. Specifically, the reduced downsampling rate in MV3L helps preserve fine-grained spatial features such as lesion boundaries, while the optimized dilation configuration enhances the receptive field without introducing excessive computational cost. This design enables MV3L to maintain a strong semantic understanding of lesion structures while retaining edge-level detail, thus achieving a favorable balance between semantic abstraction and spatial resolution.

As shown in **Table 9**, MV3L achieved the highest segmentation accuracy (MIoU = 80.08%, MPA = 89.66%) while significantly reducing computational cost (FLOPs = 47.02 G, Params = 4.651 M) compared to ResNet50, Xception, and MobileNetV2. These findings highlight MV3L’s suitability for field deployment, where lightweight yet accurate models are essential.

**Table 9:** Comparative experiments across different models.

Model	Backbone	MIoU /%	MPA /%	Flops /G	Params /M
DeepLabv3+	ResNet50	79.8	89.63	113.27	27.822
	Xception	79.34	88.96	141.21	42.181
	MV3L	80.08	89.66	47.024	4.651
	MobilenetV2	78.58	88.3	48.883	3.856
Unet	ResNet50	79.34	88.96	184.2	43.933
PSPNet	ResNet50	75.24	87.41	118.43	46.708
HRNetV2	Hrnetv2_w32	78.26	88.28	90.972	29.54
Segformer	B0	80.07	90.24	13.562	3.715
**MMPC-DeepLabv3+**	**MV3L**	**81.23**	**89.79**	**9.695**	**3.556**

MMPC-DeepLabv3+ represents the model with the best performance among all the compared models. MV3L represents the Backbone used by the model with the best performance. 81.23 value represents the best performance of the MIou metrics. 89.79 value represents the best performance of the MPA metrics. 9.695 value represents the lowest Flops among all the comparison models. 3.556 value represents the lowest Params among all the comparison models.

Importantly, the observed performance gains are primarily attributable to the structural design of MV3L rather than external training factors such as hyperparameter tuning. The reduction in downsampling enhances spatial fidelity, while the dilation configuration improves contextual awareness—both achieved with minimal computational overhead. That MV3L surpasses deeper (e.g., Xception) and more compact (e.g., MobileNetV2) networks in accuracy while maintaining a lower parameter count and FLOPs strongly supports the claim that its superior performance stems from architectural innovation rather than experimental bias.

Furthermore, **Table 9** presents a broader comparison of the MMPC-DeepLabv3+ model against mainstream segmentation networks. When compared with DeepLabv3+ variants and other models including Unet, PSPNet, and HRNetV2, MMPC-DeepLabv3+ achieves the highest segmentation accuracy in terms of both MIoU (81.23%) and MPA (89.79%), while also demonstrating the lowest FLOPs (9.695 G) and Params (3.556 M). Compared to the Segformer model, MMPC-DeepLabv3+ shows a 1.16% gain in MIoU and a slight 0.45% drop in MPA, but only consumes 71.49% of Segformer’s FLOPs and has fewer parameters by 0.159 M—demonstrating a superior balance between accuracy and computational efficiency. This 28.51% reduction in FLOPs is particularly impactful in real-world agricultural deployments, where models are expected to operate on mobile or embedded devices with stringent hardware limitations. Even marginal savings in FLOPs can directly translate to reduced power consumption, faster inference, and lower latency—all of which are critical for enabling real-time disease detection in the field. Thus, MMPC-DeepLabv3+ not only slightly surpasses Segformer in segmentation accuracy but does so with significantly lower computational demand, making it more practical for low-resource precision agriculture scenarios.

To ensure a fair and unbiased comparison, all baseline models, including U-Net, PSPNet, HRNetV2, DeepLabv3+ variants, and Segformer, were trained under the same experimental setup as the proposed MMPC-DeepLabv3+. This included consistent data preprocessing steps (such as image resizing and normalization), the same training-validation split (9:1), the use of the identical data augmentation pipeline applied only to the training set, and unified training hyperparameters (optimizer, learning rate, batch size, number of epochs, and loss function). By maintaining identical training conditions, this study ensures that performance differences among models are attributable solely to architectural variations rather than inconsistencies in training strategy or dataset processing.

#### 3.5.2 Precision-based analysis and disease-specific challenges

In addition to the overall performance metrics, a detailed class-wise precision comparison is presented in **Table 10**. The proposed MMPC-DeepLabv3+ achieves the highest mean precision (89.43%) among all models, demonstrating robust performance across all lesion categories. Notably, MMPC-DeepLabv3+ exhibits substantial advantages in the Brown Spot (75.97%) and Bacterial Blight (89.01%) classes, which are commonly prone to misclassification due to visual similarities with other lesions.

**Table 10:** Different Model Precision Comparison.

Model	Background/%	Healthy Leaf/%	Brown Spot/%	Rice Blast/%	BacterialBlight/%	mPricision/%
DeepLabv3+	99.08	94.94	71.87	83.96	86.07	87.18
Unet	99.13	94.97	75.05	80.02	86.98	87.39
PSPNet	99.16	95.07	56.87	81.89	85.14	83.63
HRNetV2	98.99	96.18	63.18	88.17	88.95	87.09
Segformer	99.01	95.96	72.99	87.1	86.11	88.23
**MMPC-DeepLabv3+**	99.09	96.16	75.97	87.9	89.01	89.43

MMPC-DeepLabv3+ denotes the improved model proposed in this study.

For instance, Brown Spot and Rice Blast lesions often appear as circular or elliptical necrotic areas, especially during early infection stages or under suboptimal imaging conditions, such as uneven lighting. Similarly, the pale, elongated streaks caused by Bacterial Blight can resemble senescence symptoms or mechanical injuries on healthy leaves, leading to increased false positives. These overlapping visual features pose significant challenges for segmentation models that lack fine boundary perception or texture differentiation capabilities.

This challenge is evident in baseline models such as PSPNet and HRNetV2, which recorded relatively low precision for Brown Spot (56.87% and 63.18%, respectively). Their traditional fusion mechanisms and limited contextual adaptability reduce their ability to distinguish between diseases with subtle morphological differences.

In contrast, MMPC-DeepLabv3+ integrates the MSDE module to enhance multi-scale boundary detail and texture extraction, while the PGFF feature fusion mechanism promotes efficient semantic interaction between high- and low-level features. These design choices directly improve the model’s capacity to differentiate visually similar disease symptoms, particularly inss complex, multi-disease field scenarios. Although Segformer also performs competitively with a mean precision of 88.23%, its accuracy in categories with high inter-class ambiguity—such as Bacterial Blight (86.11%) and Brown Spot (72.99%)—remains slightly inferior to MMPC-DeepLabv3+.

Overall, this class-level precision analysis highlights the discriminative strength and robustness of MMPC-DeepLabv3^+^ under practical agricultural conditions. By effectively handling inter-class feature overlaps, the model enhances reliability in field-level disease diagnosis and contributes to more accurate, data-driven decision-making for precision crop management.

### 3.6 Visual comparison of segmentation effects of different models

To visually demonstrate the performance of MMPC-DeepLabv3+, the segmentation effects of the MMPC-DeepLabv3+, DeepLabv3+, HRNetV2, PSPNet, and Unet were compared under complex scenarios such as background interference, bright light environments, and blade overlap. In addition, the performance of segmenting small targets as well as the accuracy and fineness during boundary segmentation were also compared.

#### 3.6.1 Comparison of model segmentation effects in complex scenes

**Figure 11:**
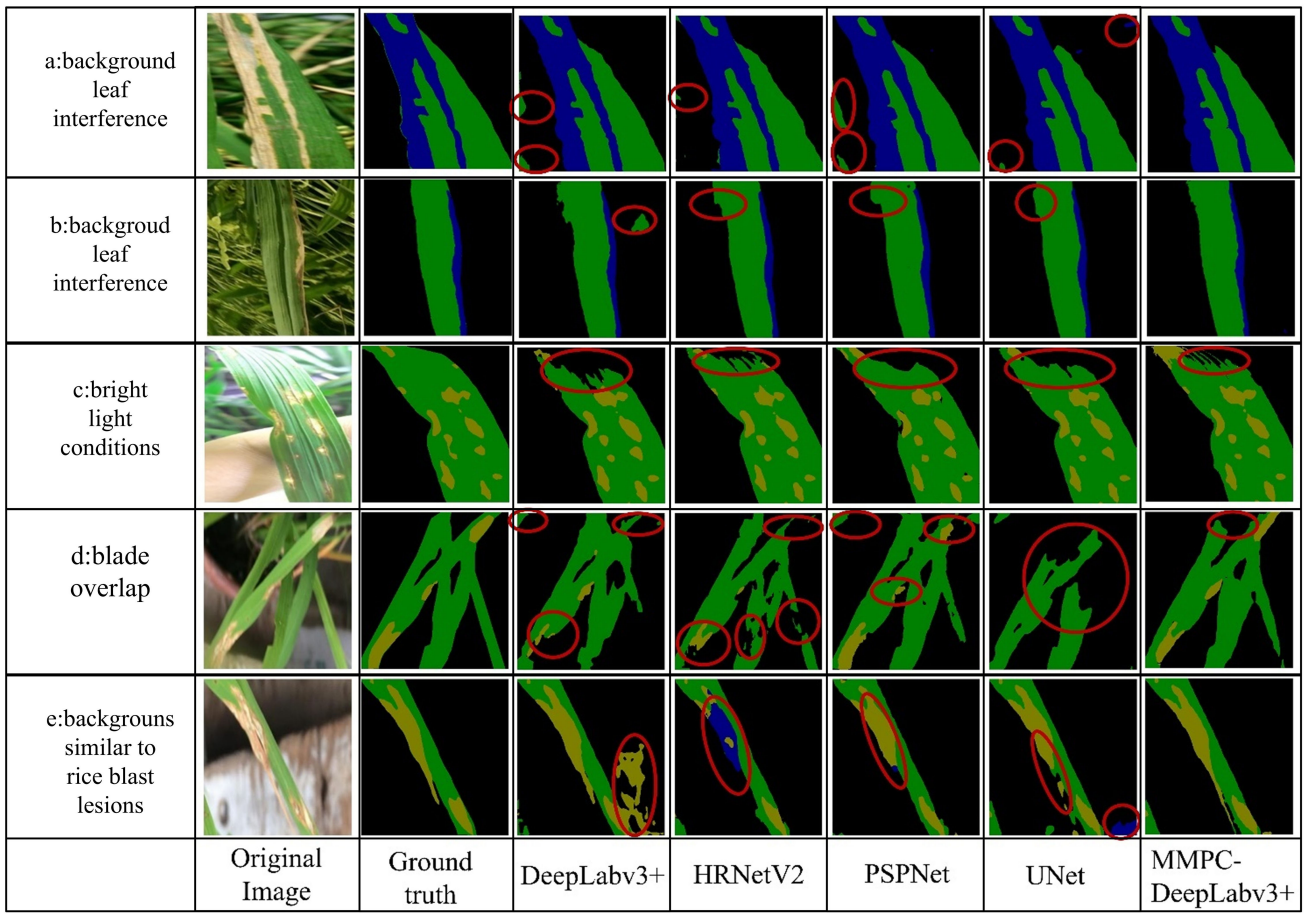
Comparison of model segmentation performance in complex scenes. Where **(a, b)** represent background leaf interference; **(c)** corresponds to bright light conditions; **(d)** indicates blade overlap; and **(e)** denotes backgrounds similar to rice blast lesions.

**Figure 11** illustrates a visual comparison of segmentation results among five models—MMPC-DeepLabv3+, DeepLabv3+, HRNet, PSPNet, and UNet—under four challenging scenarios: background leaf interference, bright light conditions, blade overlap, and backgrounds similar to rice blast lesions. Specifically, Scenario a and Scenario b both involve complex background interference, but Scenario a emphasizes spatial location distractions, while Scenario b highlights color confusion between the background and leaf textures.

From left to right, each image group displays the original one, its corresponding color-mapped label image (ground truth), and the segmentation outputs of DeepLabv3+, HRNet, PSPNet, UNet, and MMPC-DeepLabv3+. In the segmentation maps, black represents the background, yellow denotes rice blast, and blue indicates bacterial leaf blight.

In the background leaf interference scenario (Scene a), all models except MMPC-DeepLabv3+ were distracted by the overlapping rice leaves in the lower-left region, failing to focus on segmenting the primary leaf and its bacterial leaf blight lesions. In the background color-texture confusion (Scene b), all models segmented the bacterial leaf blight on the main leaf in the figure relatively accurately. However, when segmenting healthy leaves, the comparative models failed to accurately distinguish other green leaves in the background.

Under bright light conditions (Scene c), bright spots of reflection on the upper leaf surface interfered with the models’ recognition of this area, resulting in an under-segmentation phenomenon for all models in this area and failing to accurately classify the specific category of this area. Nevertheless, the under-segmented area of the MMPC-DeepLabv3+ model when dealing with this interference was the smallest, showing better robustness compared to other models.

In the scenario of blade overlap (scenario d), the overlapping leaves increased the difficulty of segmentation. The other four models failed to clearly segment the rice blast spots and had under-segmentation to varying degrees. In contrast, the MMPC-DeepLabv3+ model segmented the lesion area more accurately, and the under-segmentation phenomenon was relatively mild.

In the scenario where the background is similar to rice blast (scenario e), the background is very similar to rice blast, and the segmentation effects of the comparative models are all unsatisfactory: The DeepLabv3+ mistakenly identified a part of the background as rice blast; HRNetV2 misjudged most of the rice blast areas on the main leaf as bacterial leaf blight; although the segmentation effect of PSPNet was relatively better, it still misjudged a small part of the rice blast areas as bacterial leaf blight; and Unet misidentified the background at the lower right of the image as bacterial leaf blight. On the contrary, the MMPC-DeepLabv3+ model did not misjudge the background and segmented the lesion area on the leaf relatively accurately.

#### 3.6.2 Comparison of small target and boundary segmentation effects

**Figure 12:**
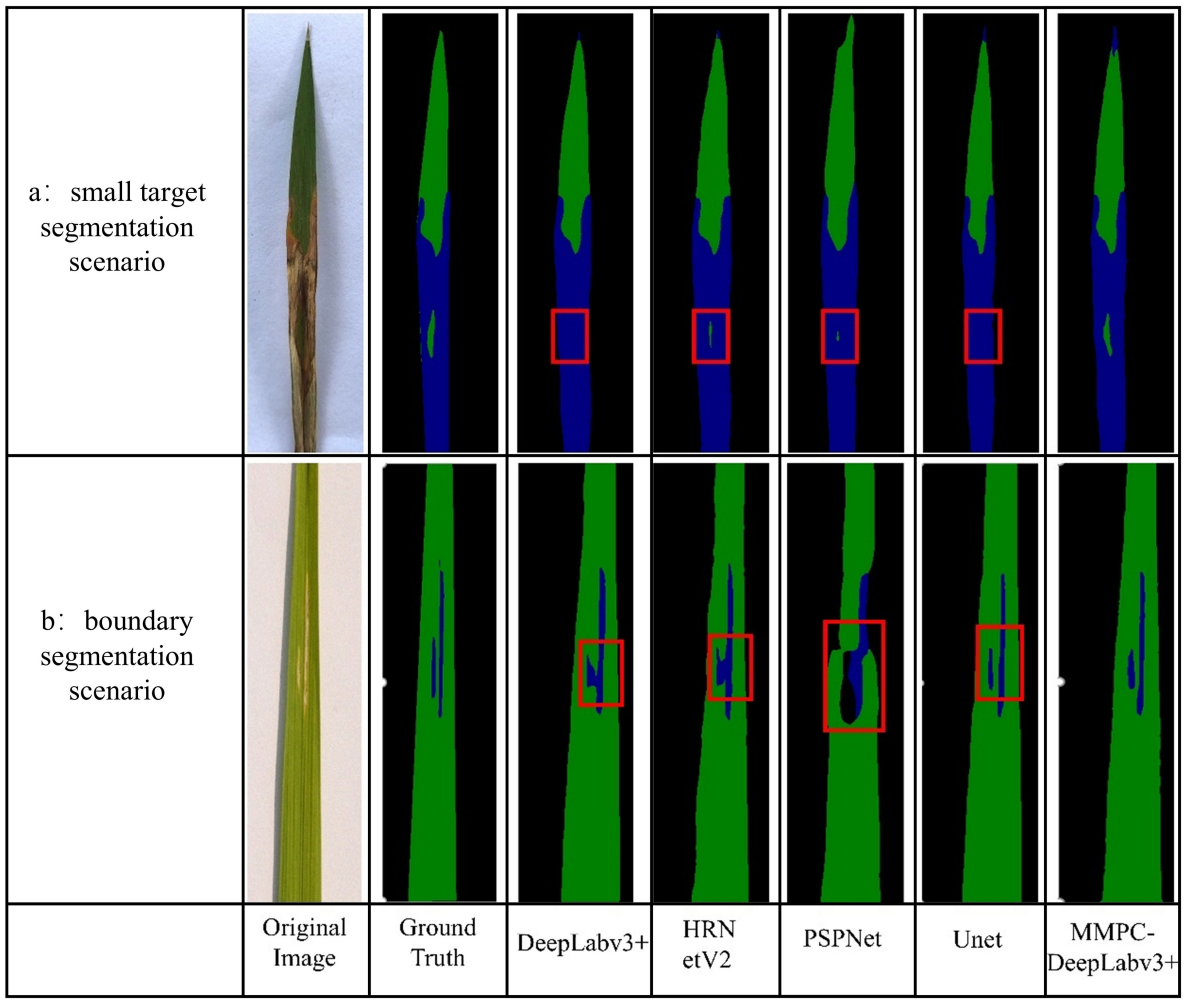
Comparison of small object and boundary segmentation effects. The red boxes delineate the segmentation performance of comparative models in challenging scenarios: **(a)** small object segmentation and **(b)** boundary delineation tasks.

As illustrated in **Figure 12**, the image comparison showcases each performance of the model in small-object segmentation and boundary delineation. In Scenario a (small target segmentation scenario), the target region is a tiny healthy leaf patch surrounded by bacterial leaf blight lesions. Visual analysis reveals that both DeepLabv3+ and UNet failed to detect this small region, while MMPC-DeepLabv3+, HRNetV2, and PSPNet successfully identified it—though with varying segmentation quality. Notably, only MMPC-DeepLabv3+ achieved a relatively complete segmentation of the small healthy area, whereas the other two models (HRNetV2 and PSPNet) only captured partial regions.

In Scenario b (boundary segmentation scenario), the lesion area consists of two vertically parallel and independent regions. Although the DeepLabv3+ and HRNetV2 models recognized the lesion regions of bacterial leaf blight, they exhibited boundary confusion at the proximity of the nearby patches, failing to clearly separate the adjacent lesions. In contrast, both MMPC-DeepLabv3+ and UNet successfully distinguished the two lesion areas, producing comparatively sharp segmentation boundaries.

## 4 Discussion

For a rice leaf lesion segmentation model intended for practical field applications, achieving an effective balance between segmentation accuracy and computational complexity is essential. In the standard DeepLabv3+, relying solely on the backbone network for 4× downsampling to extract low-level features is insufficient for tasks highly sensitive to fine details, such as rice lesion segmentation. The proposed MSDE module incorporates Haar wavelet downsampling, decomposing 2D images into primary information and directional details information (horizontal, vertical, diagonal). This innovation not only performs downsampling but also decouples one high-frequency component and three low-frequency components (Waqas Ahmed et al., 2025). Based on this, the HWD module can achieve lossless encoding of features while performing downsampling. As a result, integrating the MSDE module into MMPC-DeepLabv3+ significantly improves segmentation performance for lesion boundaries and small-scale lesions.

On the other hand, the computational cost of the model is also a key consideration in this study. Replacing the backbone of the model with the optimized lightweight network MV3L not only takes advantage of the powerful feature extraction ability of MV3L (Nuanmeesri, 2025) but also reduces the Params and the Flops of MMPC-DeepLabv3+. However, this is not sufficient for full application in the rice field environment with limited computing resources. Through research, it is found that the feature fusion strategy of DeepLabv3+ imposes a relatively large computational burden on feature integration during the two operations of 4× upsampling and channel stacking.

Therefore, this study proposes the PGFF module for the rice leaf disease spot segmentation task. PagFM reduces the dimension of the input features through 1×1 convolution, significantly reducing the Params and the Flops. At the same time, after the initial fusion of PagFM, feature fusion is carried out through lightweight ghost convolution, further improving the computational efficiency. Secondly, the weight coefficients of low-level features and high-level features allow MMPC-DeepLabv3+ to make adaptive adjustments based on the semantic contributions of features, better bridging the semantic gap between features at different levels.

Finally, adding the CA mechanism before the high-level and low-level features output by the MMPC-DeepLabv3+ model further enhances the feature representation ability of MMPC-DeepLabv3+ and improves the robustness in complex segmentation scenarios (Liu et al., 2024). The ablation experiments show that the improvements made in this study can enable the model to have higher segmentation accuracy while significantly reducing the Flops and Params of the model.

In addition, Tassis et al. (2021) used single-stage models of PSPNet and U-Net to segment coffee leaf disease spots in an outdoor environment. Among them, the Params and Flops of PSPNet reached 49.06 M and 61.47 G, respectively, and the Params and Flops of U-Net also reached 31.03 M and 261.84 G. Compared with them, the Params and Flops of MMPC-DeepLabv3+ are only 7.24% and 15.77% of those of PSPNet. At the same time, compared with U-Net, the Params and Flops of MMPC-DeepLabv3+ are reduced by 88.52% and 96.29%, respectively. Wang et al. (2021) utilized the two-stage segmentation model DUNet to segment cucumber disease spots in natural scenes. In this model, DeepLabv3+ was used for leaf segmentation in the first stage, and U-Net was used for disease spot extraction in the second stage. Compared with this method, the params and Flops of MMPC-DeepLabv3+ are reduced by 30.22 M and 278.48 G, respectively. MMPC-DeepLabv3+ has a smaller model size and computational cost, making it more suitable for deployment in field environments with limited computing resources. Similarly, Yang et al. (2024) introduced LT-DeepLab for segmenting Zanthoxylum bungeanum leaves, achieving 76.58% MIoU and 86.02% MPA, with 54.68M parameters and 245.76G Flops. In contrast, our proposed MMPC-DeepLabv3^+^ achieves superior segmentation performance (MIoU = 81.23%, MPA = 89.79%) with significantly lower computational cost (3.556M parameters, 9.695G FLOPs), demonstrating its enhanced efficiency and suitability for mobile deployment in real-world agricultural environments.

To ensure practical applicability, the proposed MMPC-DeepLabv3+ model was designed with lightweight modules to reduce computational cost, enabling deployment on edge devices such as mobile phones, tablets, or embedded systems in field environments. The enhanced segmentation of small lesions and boundary details can assist agricultural practitioners in early disease detection and management decisions. Furthermore, the output segmentation masks can be integrated into mobile applications or IoT-based agricultural monitoring systems to provide real-time visual feedback and automated disease assessment, thus facilitating precise and timely interventions in crop management.

Although MMPC-DeepLabv3+ achieved promising results, several limitations should still be acknowledged to guide future improvements. First, although the dataset was expanded through augmentation, the number of original images was relatively limited and may not fully capture the diversity of real-world field conditions. Second, the study focused only on three rice leaf disease types, excluding other relevant conditions such as sheath blight or tungro virus, which limits the scope of practical applicability. Third, the robustness of the model across different imaging devices, resolutions, and environmental variations was not systematically evaluated, as most images were collected under relatively controlled conditions.

In future work, efforts will be made to expand the dataset by collecting a greater number of original field images from diverse geographic regions and environmental settings. This will help improve the generalizability and robustness of the model in real-world scenarios, addressing the current limitation of limited image diversity. Additionally, the range of disease types will be broadened to include other important rice leaf conditions such as sheath blight and tungro virus. This extension will enhance the applicability of the model across different agronomic regions and disease spectrums.

Furthermore, although the current dataset already includes images with natural shadows, partial occlusions, and environmental noise (e.g., dust, leaf damage), future work will focus on conducting controlled robustness evaluations. This includes applying artificial adversarial perturbations (such as shadow simulation, occlusion overlays, and Gaussian distortions) to systematically assess the model’s stability under extreme visual disturbances. These tests will help quantify MMPC-DeepLabv3+’s resilience to real-world image interference and guide the development of further enhancements to its feature encoding and fusion strategies.

Additionally, the dataset will be enriched with images of lesions at different developmental stages to capture intra-class variation, which is essential for modeling disease progression over time. Lastly, multimodal learning approaches will be explored by integrating auxiliary data such as crop growth stages, soil properties, and environmental factors, aiming to enable a more comprehensive and accurate quantitative assessment of disease severity.

## 5 Conclusions

In this study, MMPC-DeepLabv3+ was proposed as an improved lesion segmentation model based on DeepLabv3+. A series of lightweight and effective modules—including MV3L for backbone replacement, MSDE for multi-scale detail enhancement, PGFF for progressive feature fusion, and CA for attention refinement—were integrated into the model.

Through comparative experiments with DeepLabv3+, Unet, PSPNet, and HRNetV2, its effectiveness was validated. The proposed model achieved an increase of 1.89% in MIoU and 0.83% in MPA, while reducing FLOPs and Params by 93.1% and 91.6%, respectively. Better performance was observed particularly in segmenting fine boundaries and small disease targets under complex backgrounds. As a result, MMPC-DeepLabv3+ was demonstrated to be a promising solution for efficient and accurate segmentation in resource-constrained agricultural scenarios.

## Data Availability

The original contributions presented in the study are included in the article/supplementary material, further inquiries can be directed to the corresponding authors.
